# Application of PET/CT in treatment response evaluation and recurrence prediction in patients with newly-diagnosed multiple myeloma

**DOI:** 10.18632/oncotarget.11418

**Published:** 2016-08-19

**Authors:** Ying Li, Junru Liu, Beihui Huang, Meilan Chen, Xiangwen Diao, Juan Li

**Affiliations:** ^1^ Department of Hematology of The First Affiliated Hospital of Sun Yat-sen University, Guangzhou, Guangdong, China

**Keywords:** multiple myeloma, PET/CT, treatment response

## Abstract

Multiple myeloma (MM) causes osteolytic lesions which can be detected by 18F-fluorodeoxyglucose positron emission tomography/Computed tomography (18F-FDG PET/CT). We prospectively involve 96 Newly diagnosed MM to take PET/CT scan at scheduled treatment time (figure 1), and 18F-FDG uptake of lesion was measured by SUVmax and T/Mmax. All MM patients took bortezomib based chemotherapy as induction and received ASCT and maintenance. All clinical features were analyzed with the PET/CT image changes, and some relationships between treatment response and FDG uptakes changes were found: Osteolytic lesions of MM uptakes higher FDG than healthy volunteers, and this trend is more obvious in extramedullary lesions. Compared to X-ray, PET/CT was more sensitive both in discoering bone as well as extramedullary lesions. In newly diagnosed MM, several adverse clinical factors were related to high FDG uptakes of bone lesions. Bone lesion FDG uptakes of MM with P53 mutation or with hypodiploidy and complex karyotype were also higher than those without such changes. In treatment response, PET/CT showed higher sensitivity in detecting tumor residual disease than immunofixation electrophoresis. But in relapse prediction, it might show false positive disease recurrences and the imaging changes might be influenced by infections and hemoglobulin levels. Conclusion: PET/CT is sensitive in discovering meduallary and extrameduallary lesions of MM, and the 18F-FDG uptake of lesions are related with clinical indictors and biological features of plasma cells. In evaluating treatment response and survival, PET/CT showed its superiority. But in predicting relapse or refractory, it may show false positive results.

## INTRODUCTION

Multiple myeloma (MM) is a malignant plasma cell tumor that is often complicated by bone destruction in clinical practice. Typically, radiological examinations that can identify bone lesions are required by clinicians for further diagnosis, disease staging and response evaluation of MM [1–2]. In the era of new drugs and hemopoietic stem cell transplantation, clinicians are also seeking improved methods for radiological examination of MM [3]. Substantial effort has therefore gone into identifying safe, sensitive, and specific radiological imaging modalities for MM patients.

In recent years, PET/CT, using ^18^F-FDG as the tracer, has been extensively used in the diagnosis and treatment of various solid tumors and lymphoma. The new ^18^F-FDG tracer both specifically accumulates within tumors and overcomes the disadvantage of hepatic and renal toxicity that is inherent to conventional tracers, thus supports its extensive use in various patients [4–6]. Therefore, ^18^F-FDG PET/CT potentially has great diagnostic and prognostic value in the treatment of MM. Compared with conventional X-rays, systemic CT scans are more sensitive at identifying bone lesions and at reducing the misdiagnosis rate of MM; moreover, ^18^F-FDG uptake in bone lesions appears to be closely related with disease-free survival (DFS) and overall survival (OS) in MM patients [3, 7–10].

There are, however, several issues regarding the use of ^18^F-FDG PET/CT in the diagnosis and treatment of MM that require further investigation: Firstly, tumor-specific uptake of ^18^F-FDG allows clear demarcation of MM bone lesions, with the maximum standardized uptake value (SUV_max_) being the main index for measuring ^18^F-FDG uptake in bone lesions [11–12]. However, the validity of using SUV_max_ appears to be not so tumor-specific, with nervous system tumors for example showing significant inter-individual variation in the SUV_max_ of ^18^F-FDG PET/CT [13–14]. It is currently unclear whether there is a better parameter to accurately determine ^18^F-FDG uptake in MM patients. In order to overcome this dilemma, we added a new index “T/Mmax”, which alleviate the influence of background “noise” by collecting the 18F-FDG uptake by the uptake of mediastinal [13–14]. Secondly while poor molecular karyotype and high LDH in MM patients have previously been associated with high uptake of ^18^F-FDG [15]—whether such an association with other clinical characteristics exists remains unknown. Thirdly, although ^18^F-FDG uptake in lesions has been examined in relation to progression-free survival (PFS) and overall survival (OS) after treatment [3, 7–10], the relationship between post-treatment ^18^F-FDG uptake in lesions and clinical response and disease recurrence remains unknown. Here, in an attempt to determine the relationship between the radiological profile of bone lesions and clinical parameters in MM patients, we prospectively performed ^18^F-FDG PET/CT scans on 98 newly-diagnosed MM patients and followed their images changes during treatment. Up to now, 34 patients have finished their observation and we observed some interesting results through these cases, by presenting our cases, we hoped we will clarify the value of PET/CT in predicting disease recurrence.

## MATERIALS AND METHODS

### Subjects

**Figure 1:**
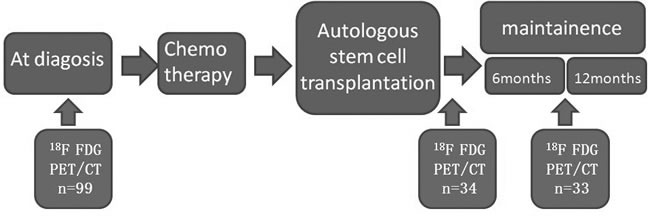
The process of the prospective design

Ninety-eight newly-diagnosed MM patients who were given standardized treatment at the First Affiliated Hospital of Sun Yat-sen University between January 2010 to June 2014 prospectively received PET/CT scans and systemic bone X-rays at the onset of disease and during treatment (Figure 1). were enrolled in this study. Staging criteria for MM were based on IMWG international diagnostic criteria for multiple myeloma [16]. PET/CT scans were also performed on age- and gender-matched healthy volunteers (5 male and 10 female subjects, median age 48, age range 36-72) (Table 1). The baseline uptake range of ^18^F-FDG in normal bones was established from these 15 volunteers and the average uptake of bone marrow is 1.65 without lesions or fractures. Disease onset data of the 98 MM patients as well as the healthy volunteer are provided in Table 1. This study was approved by the Ethics Committee at the First Affiliated Hospital of Sun Yat-sen University, and all subjects provided written informed consent for this study.

**Table 1:** Baseline information of the 98 NDMM patients at the onset of disease

Factor	N/n, (%)
Male in healthy volunteer	10/15(66.67)
Median age at PET/CT scan in healthy volunteer(yrs)	48 (36-72)
Male in MM	52/98 (53.06)
Median age at diagnosis (range) (yrs)	55 (31–72)
M protein type in MM	
IgG	51/98 (52.04)
IgA	13/98 (13.27)
IgD	5/98 (5.10)
Light chain	29/98 (32.58)
Durie Salmon stage at diagnosis in MM	
Stage 2	9/98 (9.18)
Stage 3	89/98 (90.81)
International Staging System at diagnosis in MM	
Stage 1	40/98(40.82)
Stage 2	43/98 (43.88)
Stage 3	15/8 ( 15.30)
Serum Creatinie > 2 mg/dl at diagnosis in MM	28/98 (28.57)
FISH at diagnosis in MM	
Del 17p	11/72 (15.28)
T(14:16)	0/72 (0)
T(4:14)	9/72 (12.50 )
T(11:14)	11/72 (15.28)
1q21	30/72 (41.67)
Complex karyotype or hypokaryotype in MM	21/72 (29.17)

Abbreviations: n, number of total cases; N, number of cases; %, percentage; PET/CT, positron emission tomography/Computed tomography; MM, multiple myeloma; FISH, fluorescence *in situ* hybridization; mSMART, Mayo Stratification for Myeloma And Risk-adapted Therapy.

### Therapeutic regimen, treatment response evaluation and follow-up

34 patients in observation completed chemotherapies of bortezomib and dexamethasone combined with PEGylated liposomal doxorubicin (PAD regimen). The detailed chemotherapeutic regimen was as follows: 1.3 mg/m^2^ bortezomib i.v. bolus over 3 seconds on days 1, 4, 8 and 11; 20 mg/d dexamethasone, i.v. drip on days 1-4; and 40 mg/m^2^ PAD, i.v. drip on day 4. MM patients without renal insufficiency were treated with cyclophosphamide +granulocyte colony stimulating factor for mobilization of peripheral blood hematopoietic stem cells, and collection of stem cells were performed after achieving best response, after which patients received autologous hematopoietic stem cell transplantation (ASCT). The pretreatment regimen for ASCT was 200 mg/m^2^ i.v. drip melphalan. For patients with renal insufficiency, hematopoietic stem cells were collected directly from bone marrow, and the pretreatment regimen was adjusted to 100-140 mg/m^2^ melphalan i.v. drip according to patients’ glomerular filtration rate. Maintenance therapy was initiated soon after hematopoietic recovery, which consisted of thalidomide, lenalidomide, interferon-α alone or thalidomide combined with interferon-α. MM patient responses were evaluated according to EBMT response evaluation criteria (Table 3) [17].

**Table 3:** EBMT for response

Complete response	No M-protein detected in serum or urine by immunofixation for a minimum of 6 weeks and fewer than 5% plasma cells in bone marrow
Partial response	>50% reduction in serum M-protein level and/or 90% reduction in urine free light chain excretion or reduction to <200 mg/24 h for 6 weeks
Minimal response	25–49% reduction in serum M-protein level and/or 50–89% reduction in urine free light chain excretion which still exceeds 200 mg/24 h for 6 weeks
No change	Not meeting the criteria of either minimal response or progressive disease
Plateau	No evidence of continuing myeloma-related organ or tissue damage <25% change M-protein levels and light chain excretion for 3 months
Progressive disease	Myeloma-related organ or tissue damage continuing despite therapy or its re-appearance in plateau phase >25% increase in serum M-protein level (>5 g/l) and/or >25% increase in urine M-protein level (>200 mg/24 h and/or >25% increase in bone marrow plasma cells (at least 10% in absolute terms)
Relapse	Reappearance of disease in patients previously in CR, including detection of paraprotein by immunofixation

### PET/CT

The research strictly followed the process of Figure 1. ^18^F-FDG was prepared using a GE PETtracer cyclotron and automatic synthesis system, and the radiochemical purity was > 95%. Systemic PET scans were performed with a PET/CT scanner (Gemini GXL, Philips). CT scans were used for anatomical location and decay correction. After fasting for at least 6 hours, patients were injected with ^18^F-FDG (259-444 MBq) i.v. through a three-limb tube after which patients were instructed to lie still for 60 minutes in a dark room, which was followed by a PET/CT scan after urination. PET scans included an emission and projection scan, at a rate of 4-5 minutes/bed and 3-4 minutes/bed, respectively. CT scans were performed at a voltage of 140 kV, current 200 mA, thread pitch 0.75 and the rotation time of each cycle was 0.8 s. The PET and CT images were processed using eNTEGRA workstation (Siemens, Germany).

All radioactive concentrated lesions on PET images were independently reviewed by 2 experienced nuclear medicine physicians, who were blinded to patient clinical data. If the 2 physicians disagreed on the evaluation of an image, the ^18^F-FDG PET/CT image scan was reviewed again, discussed, and analyzed until consensus was reached.

Destructive bone lesions were assessed using IMWG criteria [18], and once diagnosed, the lesions were anayalyzed by eNTEGRA workstation of its maximum ^18^F-FDG uptake. If no pathologically concentrated lesions occurred after treatment, lesions displaying concentrated ^18^F-FDG uptake prior to treatment were selected for SUV_max_ measurements. None of the patients had mediastinal disease or mediastinal tumor infiltration, and the SUV_max_ of the mediastinum was therefore measured at the same time as ^18^F-FDG uptake in normal tissues for this ^18^F-FDG PET/CT scan. T/M_max_ was defined as the ratio of SUV_max_ in lesions to SUV_max_ in the mediastinum (T/M_max_ = SUV_max_ in bone or tissue lesions / SUV_max_ in mediastinum).

### Statistical analysis

Receiver operating characteristic (ROC) curves were used to determine the accuracy, sensitivity and specificity of T/M_max_ for the detection of lesions in MM patients. Wilcoxon rank-sum test was used to analyze the difference in SUV_max_ and T/M_max_ between MM patients with different clinical characteristics, and Pearson's chi-square test was used to compare the incidence of extramedullary lesions in MM patients with different clinical characteristics. Survival analysis was performed based on PFS, OS, SUV_max_ and T/M_max_ of lesions in MM patients. PFS was defined as the time from the start of consolidation or maintenance treatment to disease progression or recurrence. OS was defined as the time from the onset of disease to the time of death from any cause. The Kaplan-Meier method was used for survival analysis. SPSS 19.0 software (SPSS Inc.; Tokyo, Japan) was used for all the statistical analyses. Results of *P* < 0.05 were deemed statistically significant.

## RESULTS

### PET/CTPET/CT characteristics in newly-diagnosed MM patients

Patients with newly diagnosed MM have higher ^18^F-FDG uptake in bone destructive lesions than healthy controls, with higher uptake in extramedullary lesions than intramedullary lesions

No statistical difference was found in age or gender composition between the 15 healthy volunteers and the 98 patients with newly diagnosed MM (*P* > 0.05). Bone SUV_max_ in the healthy volunteers was 1.70 ± 0.42 (95%CI 1.47-1.93) and bone T/M_max_ was 1.29 ± 0.27 (95%CI 1.14-1.44).

**Figure 2:**
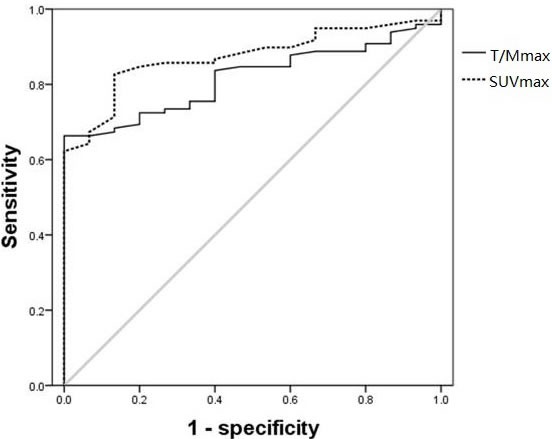
ROC curves of SUV_max_ and T/M_max_ in lesions in 98 MM patients with 15 healthy volunteers as control The accuracy, cutoff, sensitivity and specificity were 87.3%, 1.95, 80.6% and 86.7% for SUV_max_, respectively; and 82.0%, 1.69, 65.3% and 100.0% for T/M_max_, respectively.

The sensitivity and specificity of SUV_max_ and T/M_max_ in evaluating bone lesions in MM were compared using ROC curves, and both methods showed good differentiation ability between normal and pathological bone marrow (*P* < 0.001) (Figure 2). For the detection of intramedullary lesions, SUV_max_ had an accuracy of measuring bone lesions of 87.3% with a cutoff of 1.95, sensitivity of 80.6% and specificity of 86.7%; meanwhile, T/M_max_ had an accuracy, cutoff, sensitivity and specificity of 82.0%, 1.69, 65.3% and 100.0%, respectively.

Both SUV_max_ and T/M_max_ were higher in bone destructive lesions in MM patients than in healthy controls (SUV_max_: 3.20 *vs*. 1.65, *P* < 0.001; T/M_max_: 2.11 *vs*. 1.19, *P* < 0.001). 29 of 98 patients with newly diagnosed MM had extramedullary lesions (identified from PET/CT scans), and plasmacytoma was confirmed by biopsy at pathological lesions. In the 29 patients, the median SUV_max_ and median T/M_max_ was higher in extramedullary lesions than in bone destructive lesions (SUV_max_: 4.60 *vs*. 3.50, *P* = 0.024; T/M_max_: 3.21 *vs*. 2.60, *P* = 0.023).

### Increased detection of bone lesions and extramedullary lesions by PET/CT compared with systemic bone X-rays in newly diagnosed MM patients

Various bone lesions were identified in all 98 MM patients through PET/CT scans, including osteolytic changes or pathological bone fractures in 96 patients and extensive osteoporosis in 2 patients. However, when using systemic bone X-rays, only 82 patients were identified as having bone lesions, including osteolytic changes or pathological bone fractures in 77 patients and extensive osteoporosis in 5 patients. The bone lesion detection rate by PET/CT was significantly higher than by systemic bone X-ray in newly diagnosed MM patients (100.00% *vs*. 83.67%, *P* < 0.001); in particular the detection rate of osteolytic changes or pathological bone fractures (97.96% *vs*. 78.57%, *P* < 0.001).

Bone lesions in MM patients were classified as belonging to 1 of 5 areas: skull, vertebral body, thoracic bone, pelvis or 4 limbs. The cumulative range of bone lesion involvement was evaluated based on these 5 areas. The median range of bone lesion involvement was 4 areas/case by PET/CT, compared with 3 areas/case by systemic bone X-ray, and this difference was significant (*P* < 0.001). Further analysis demonstrated a higher rate of bone lesion detection in the skull, vertebral body, thoracic bone and pelvis by PET/CT compared with systemic bone X-ray (Table 2, *P* < 0.05). There was however no significant difference in the detection rate of lesions in the 4 limbs between the 2 radiological methods (Table 2, *P* = 0.096).

**Table 2:** Comparison of the lesion detection rate in different bone areas

	PET/CT	Systemic bone X-ray	*P*
Skull	68.37%	54.08%	0.020
Vertebral body	86.73%	51.02%	<0.001
Thorax	79.59%	52.04%	<0.001
Pelvic	80.61%	44.90%	<0.001
Extremities	63.27%	54.08%	0.096

Twenty-nine of 98 patients with newly diagnosed MM had extramedullary lesions (identified from PET/CT scans), however, using systemic bone X-rays, the extramedullary lesions were identified only in 6 patients, which demonstrates the significantly higher detection rate of extramedullary lesions by PET/CT than X-rays (29.59% *vs*. 6.12%, *P* < 0.001). The detection of parabone soft tissue masses in these 29 patients was also significantly higher by PET/CT where 24 parabone soft tissue masses were detected compared with only 6 by systemic bone X-rays (24.49% *vs*. 6.12%, *P* < 0.001); further, the 5 extramedullary lesions that were non-parabone masses (3 abdominal masses, 1 muscle tissue mass in 4 limbs, 1 joint soft tissue mass), could not be detected by systemic bone X-ray (5.10% *vs*. 0.00%, *P* = 0.012).

### Correlation between ^18^F-FDG uptake and clinical parameters in patients with newly diagnosed MM

Subgroup analysis was performed to evaluate ^18^F-FDG uptake in bone destructive lesions in relation to prognostic and clinical parameters at the onset of disease in the 98 patients with newly diagnosed MM (Table 3). Our analysis revealed that at the onset of disease, patients < 50 years of age, patients have free immunoglobulin light chains circulating in serum, patients with extramedullary infiltration, patients with hemoglobuliln (Hb) ≥ 120 g/L, and patients with increased LDH had higher SUV_max_ and T/M_max_ scores than the relative groups (*P* < 0.05). On the other hand, The SUV_max_ and T/M_max_ has no statistically significant differences between different M protein level and β2-MG levels groups, though M protein level and β2-MG levels were believed closely connected with tumor burden.

Patients with lower hemoglobin levels had lower SUV_max_ and T/M_max_ scores (*P* < 0.01). Patients with Hb ≤ 60 g/L had higher levels of M protein, β2-MG, and LDH than those with Hb > 60 g/L (*P* < 0.01), indicating increased tumor burden in MM patients with severe anemia. Based on the previously reported impact of hemoglobin levels on the SUV of lesions detected by ^15^O oxyhemoglobin PET/CT [19], our findings suggested that hemoglobin levels might influence ^18^F-FDG uptake and present false positive results in PET/CT-based evaluation in MM patients. Thus patients were grouped based on their hemoglobin levels at the onset of disease, while excluding patients with Hb ≤ 60 g/L. Each clinical parameter indicative of prognosis at the onset of disease was then assessed in relation to ^18^F-FDG uptake in bone destructive lesions in the 92 patients with Hb > 60 g/L (Table 4). At the onset of disease, patients < 50 years of age, patients have free immunoglobulin light chains circulating in serum, patients have extramedullary infiltration, patients have higher M protein levels, patients have > 20% plasma cells in the bone marrow smear, β2-MG > 3.5 mg/L, hypercalcemia and increased LDH had higher SUV_max_ and T/M_max_ scores (*P* < 0.05). Therefore, after excluding the influence of severe anemia, SUV_max_ and T/M_max_ in bone lesions by PET/CT were correlated with multiple parameters indicative of tumor burden in patients with newly diagnosed MM.

**Table 4:** The relationship between clinical characteristics and ^18^F-FDG uptake in lesions in 92 cases of newly-diagnosed MM patients with Hb>60g/L

Factor	Bone lesion area (Median, Quartile)	SUV_max_ (Median, Quartile)	T/M_max_ (Median, Quartile)	Extramedually rate(%)
Sex				
Male (n=49)	4.00(3.00-5.00)	3.30(2.35-5.00)	2.27(1.47-3.23)	30.61
Female (n=43)	4.00(2.00-5.00)	3.20(2.00-4.50)	2.11(1.62-2.69)	32.56
Age				
<50yrs (n=35)	4.00(3.00-5.00)	4.30(2.50-5.90)*	2.53(1.82-4.25)*	31.43
≥50yrs (n=57)	5.00(3.00-5.00)	2.70(2.15-4.00)	2.08(1.42-2.61)	31.58
DS stage				
Stage 2 (n=9)	1.00(1.00-2.00)**	2.00(1.65-4.10)*	1.70(1.39-2.40)	11.11
Stage 3 (n=83)	5.00(3.00-5.00)	3.30(2.20-5.00)	2.21(1.58-3.05)	33.73
ISS stage				
Stage 1 (n=40)	5.00(3.25-5.00)	3.30(1.92-5.14)	2.56(1.53-4.10)	45.00
Stage 2 (n=41)	4.00(3.00-5.00)	2.90(2.35-4.50)	2.10(1.47-2.83)	24.39
Stage 3 (n=11)	4.00(2.00-5.00)	3.90(3.20-4.80)	2.28(1.73-2.91)	9.09**
M protein type				
IgG (n=48)	4.00(3.00-5.00)	2.68(2.13-4.18)	1.78(1.37-2.59)	33.33
IgA (n=11)	4.00(3.00-5.00)	2.70(2.00-3.20)	2.20(1.68-2.50)	18.18
Light chain (n=29)	5.00(3.00-5.00)	5.00(2.95-6.15)**	2.60(2.02-3.99)**	37.93
IgD (n=4)	3.50(1.50-4.75)	3.43(3.01-6.43)	2.83(2.23-3.88)	0.00
Amylodosis				
Yes (n=5)	3.00(1.50-4.50)	2.60(1.95-3.58)	1.73(1.06-3.28)	0.00**
No (n=87)	4.00(3.00-5.00)	3.20(2.20-5.00)	2.20(1.58-3.03)	33.33
Extramedual lesion				
Yes (n=29)	5.00(3.50-5.00)**	3.50(2.55-5.75)*	2.60(1.93-4.60)**	100.00**
No (n=69)	4.00(3.00-5.00)	3.20(2.10-4.50)	2.08(1.37-2.63)	0.00
M protein				
high level (n=24)	4.00(3.00-5.00)	3.80(2.55-5.49)*	2.59(2.10-4.23)*	37.50
low level (n=68)	5.00(3.00-5.00)	2.90(2.00-4.45)	2.08(1.48-2.68)	29.41
Plasma cell percentage				
>20% (n=46)	5.00(3.00-5.00)	3.75(2.15-5.98)**	2.42(1.53-4.20)**	36.96
≤20%(n=46)	4.00(3.00-5.00)	2.70(2.20-4.23)	2.08(1.57-2.65)	26.09
β2-MG				
<3.5mg/L (n=57)	4.00(3.00-5.00)	2.85(1.99-4.50)*	2.12(1.37-3.35)*	35.09
≥3.5mg/L (n=35)	5.00(3.00-5.00)	4.50(2.50-7.20)	2.92(1.67-5.25)	25.71
Renal impairment				
Yes (n=22)	4.00(3.00-5.00)	3.85(2.70-4.85)	2.33(1.90-2.75)	22.73
No (n=70)	4.00(3.00-5.00)	2.90(2.08-5.03)	2.11(1.47-3.15)	34.29
Hypercalcemia				
Yes (n=14)	5.00(3.75-5.00)	4.15(3.23-5.95)*	2.65(2.11-2.98)*	35.71
No (n=78)	4.00(3.00-5.00)	3.05(2.08-4.58)	2.09(1.45-3.04)	30.77
Hypoalbuminemia				
Yes (n=36)	4.00(2.25-5.00)	3.20(2.28-4.43)	2.08(1.47-2.88)	19.44*
No (n=56)	5.00(3.00-5.00)	3.40(2.00-5.14)	2.29(1.61-3.05)	39.29
LDH				
High (n=23)	5.00(3.00-5.00)	4.20(2.70-6.30)*	2.50(1.67-5.25)*	43.48
Normal (n=69)	4.00(3.00-5.00)	2.90(2.10-4.50)	2.08(1.42-2.83)	27.54

Footnote: *: *P*<0.05 between groups, **: *P*<0.01 between groups.

### Correlation between ^18^F-FDG uptake and molecular biological and cytogenetic profiles in patients with newly-diagnosed MM

**Figure 3:**
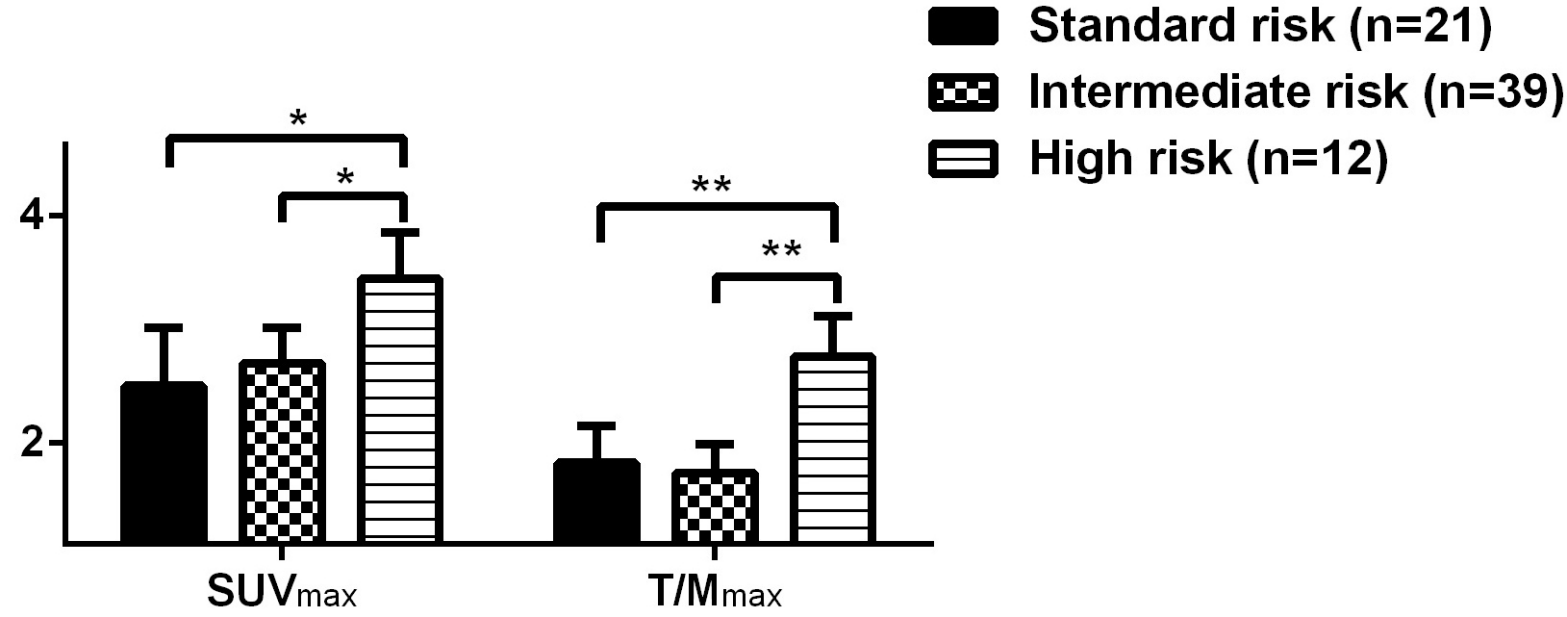
Comparison of ^18^F-FDG uptake in patients with newly-diagnosed MM at different molecular biological risks

We performed CD138 magnetic bead sorting, FISH, and chromosome karyotype analysis on bone marrow cells at the onset of disease for 72 patients to determine their molecular and cytogenetic profiles. A subgroup analysis was performed comparing the molecular biological profiles in relation to ^18^F-FDG uptake in bone destructive lesions at the onset of disease (Table 5). Patients with p53 mutations, hypoploid, or complicated karyotypes had higher T/M_max_ scores in bone lesions (*P* = 0.002, *P* = 0.031, respectively), and patients with p53 gene mutations had a higher incidence of extramedullary lesions than patients with normal p53 status (*P* < 0.001). Moreover, patients with t(11;14) chromosomal translocation gene mutations had lower SUV_max_ scores in bone lesions (*P* = 0.017), and none of these patients had extramedullary lesions. Overall, high-risk MM patients—defined by specific molecular (p53 mutations) and cytogenetic (hypoploid or complicated karyotypes) risk markers—had higher T/M_max_ (*P* < 0.01, Figure 3), and a higher incidence of extramedullary lesions (75.00% *vs*. 18.33%, *P* < 0.01).

**Table 5:** The relationship between molecular biology and cytogenetic changes and the ^18^F-FDG uptake in lesions in 72 cases of newly diagnosed MM patients

Factor	Bone lesion area (Median, Quartile)	SUV_max_ (Median, Quartile)	T/M_max_ (Median, Quartile)	Extramedually rate(%)
P53				
Positive (n=11)	5.00(3.00-5.00)	3.40(2.70-6.30)	2.62(2.08-5.25)**	72.73**
Negative (n=61)	4.00(2.00-5.00)	2.70(2.00-4.35)	1.73(1.37-2.40)	19.67
T(4:14)				
Positive (n=9)	5.00(3.00-5.00)	2.90(2.45-4.15)	1.71(1.64-2.24)	33.33
Negative (n=63)	4.00(3.00-5.00)	2.90(2.00-4.50)	2.07(1.47-2.63)	26.98
T(11:14)				
Positive (n=11)	3.00(2.00-4.00)*	2.50(1.60-3.90)	1.67(1.06-2.63)	0.00**
Negative (n=61)	5.00(3.00-5.00)	2.90(2.20-4.50)	2.08(1.57-2.56)	32.79
1q21				
Positive (n=30)	5.00(3.00-5.00)	2.70(1.90-4.28)	1.98(1.59-2.32)	26.67
Negative (n=42)	4.00(2.75-5.00)	3.20(2.15-4.63)	2.07(1.45-2.94)	28.57
karyotype				
Complex karyotype or hypodiploidy (n=21)	3.00(3.00-5.00)	3.80(2.63-5.00)	2.28(1.84-2.98)*	28.57
Normal (n=50)	5.00(3.00-5.00)	2.70(1.90-4.23)	1.76(1.36-2.47)	26.00
Trisomies (n=1)	3.00	1.90	1.58	100.00

Footnote: *: *P*<0.05 between groups, **: *P*<0.01 between groups.

### The value of PET/CT in MM treatment response evaluation

### Decreased uptake of ^18^F-FDG in lesions of MM patients with continued treatment

**Figure 4:**
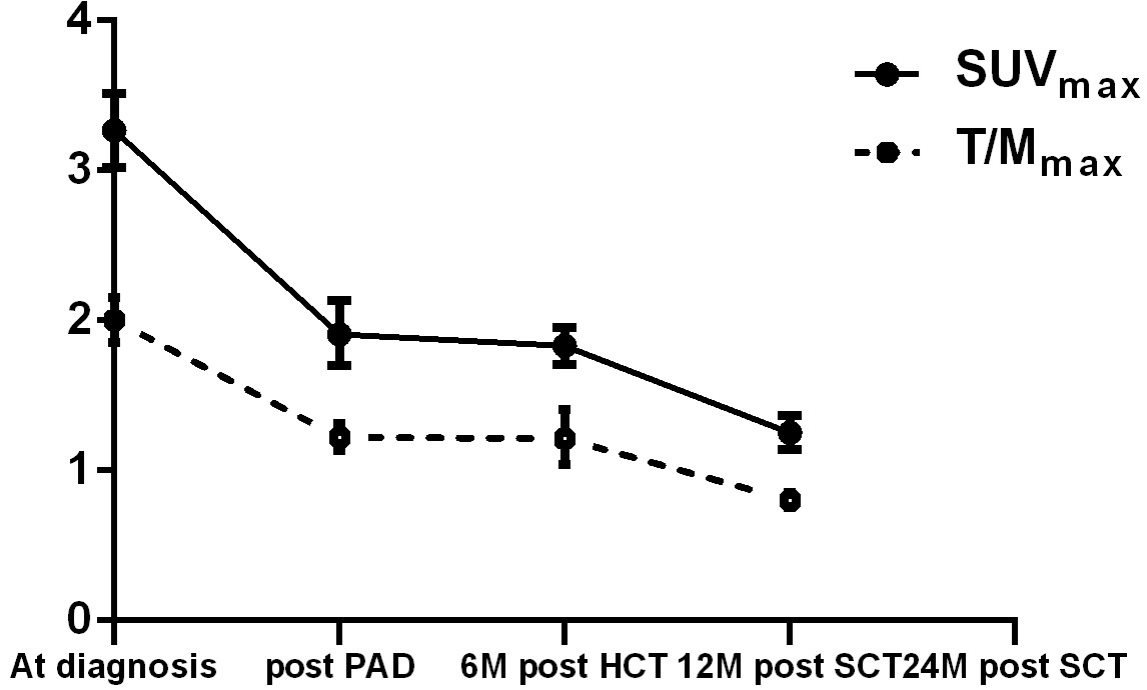
Dynamic changes in the uptake of ^18^F-FDG in lesions during treatment in the 34 MM patients

**Figure 5:**
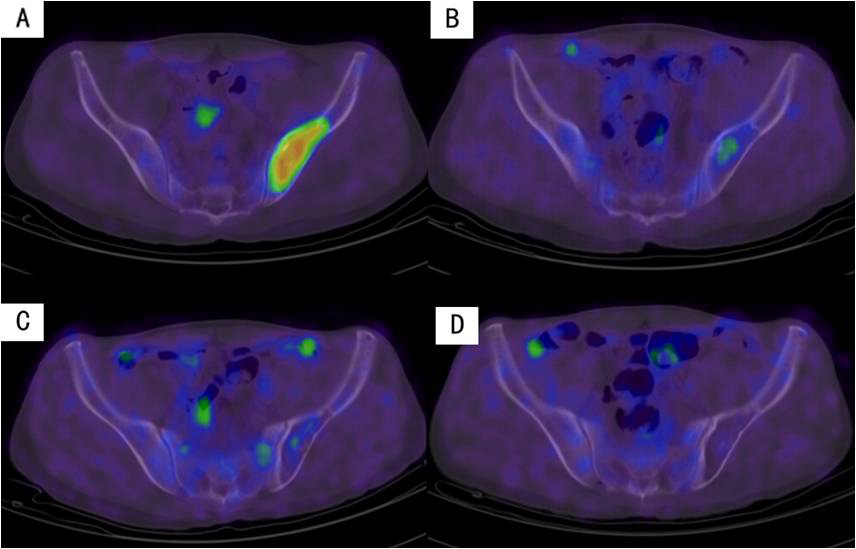
Decreased uptake of ^18^F-FDG in the iliac bone with continued treatment in an MM patient **A**. On diagnosis. **B**. After PAD regimen. **C**. Six months after transplantation. **D**. Twelve months after transplantation.

Changes in ^18^F-FDG uptake on PET/CT was dynamically analyzed prior to, after PAD regimen treatment, and within 6 and 12 months after ASCT in 34 MM patients. With continued treatment ^18^F-FDG uptake tended to decrease (Figure 4), even in certain MM patients who had achieved CR in the early part of their treatment (Figure 5).

### Correlation between ^18^F-FDG uptake in lesions and clinical response in MM patients

**Figure 6:**
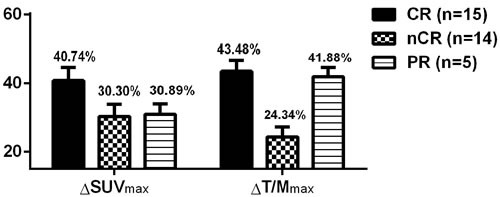
Comparison of the decrease in ^18^F-FDG uptake after chemotherapy in MM patients with different responses

Of the 34 patients receiving the PAD regimen, 15 achieved CR after chemotherapy, 14 achieved near complete remission (nCR) and 5 achieved partial remission (PR). The decrease in ^18^F-FDG uptake was more pronounced in MM patients with CR after chemotherapy compared with the rest of the patients, but this difference was not statistically significant (*P* > 0.05, Figure 6).

A subgroup analysis was performed based on the decrease in ^18^F-FDG uptake after chemotherapy in MM patients, and there was no significant difference in ^18^F-FDG uptake in patients with different responses (Table 3).

In 5 of the 15 MM patients with CR after chemotherapy, ^18^F-FDG uptake in lesions was still not reduced to normal levels, and in 4 of these 5 patients it was reduced to normal half a year after ASCT; the remaining patient had IgD-κ MM and a p53 gene mutation detected at the onset of disease and this patient was therefore given thalidomide and interferon for maintenance therapy after transplantation, and ^18^F-FDG uptake was reduced to normal one year after ASCT.

### Correlation between ^18^F-FDG uptake, OS and PFS in MM patients after chemotherapy

**Figure 7:**
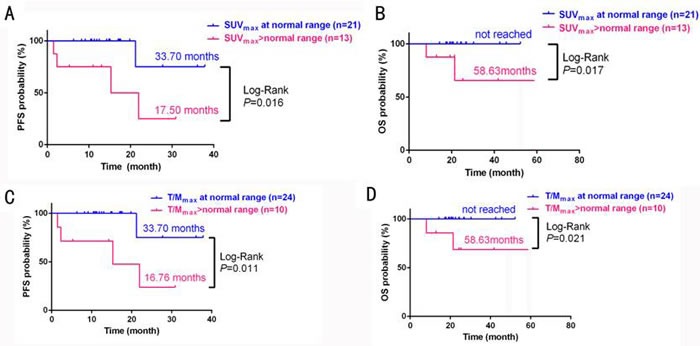
Survival analysis in MM patients who had normal or above-normal SUV_max_ (A: PFS, B: OS); or T/M_max_ (C: PFS, D: OS) at end of PAD chemotherapy treatment

The median follow-up time for the 34 patients receiving the PAD regimen was 16.63 months (4.97-33.33 months), during which one patient had disease progression approximately 5.73 months after chemotherapy, and this patient died after 7.92 months. No disease progression or deaths were recorded for any of the other patients during the follow-up period. A subgroup analysis indicated that the expected median PFS and OS was longer in MM patients in whom ^18^F-FDG uptake was reduced to normal after chemotherapy (*P* < 0.05) (Figure 7).

### The value of PET/CT in MM recurrence prediction

Of the 34 MM patients receiving the PAD regimen, ^18^F-FDG uptake increased in the bone lesions of 4 patients during treatment (Table 6). Only 1 of these 4 patients had disease progression during the follow-up period.

Patient 1 had Hb of 54 g/L and a *1q21* gene mutation at disease onset. This patient achieved CR after 4 rounds of chemotherapy, and Hb increased to 120 g/L. Six months after transplantation, his Hb rise to 124 g/L and the patient was still in CR at the last follow-up date.

Patient 2 had MM recurrence and an *IgH/FGFR* gene mutation at disease onset. After 2 rounds of chemotherapy this patient achieved nCR, and the repeated ^18^F-FDG PET/CT indicated that although SUV_max_ had decreased compared with before treatment, T/M_max_ was significantly increased; no myeloma-related chemotherapy was administered after re-examination. Two weeks later, multiple subcutaneous soft tissue masses were detected and a repeated ^18^F-FDG PET/CT indicated that SUV_max_ had increased from 6.6 to 8.4 and T/M_max_ to increased from 5.5 to 7, respectively. Plasmacytoma was confirmed by biopsy. The patient was subsequently treated with the PAD chemotherapeutic regimen but did not respond well, and died 7.92 months after the onset of disease.

Patient 3 achieved nCR after chemotherapy and CR upon reexamination 1 month after ASCT. This patient had a mass sized 3 cm × 3 cm × 1.5 cm in the right elbow with surface ulceration 11 months after transplantation. The biopsy indicated inflammation, and the mass was reduced after anti-inflammatory therapy. Re-examination 12 months after ASCT showed a clinical response of CR despite increased uptake of ^18^F-FDG.

Patient 4 achieved nCR after chemotherapy and CR upon reexamination 1 month after ASCT. However, the patient discontinued treatment 5 months after transplantation due to intolerable side effects and reexamination 6 months after ASCT showed a clinical response of CR despite increased uptake of ^18^F-FDG.

**Table 6:** Clinical data in MM patients with increased uptake of ^18^F-FDG in lesions after treatment

No.	Age (yrs)	Sex	Immunological pattern	DS	ISS	Efficacy	Onset lesions SUV_max_ T/M_max_	Post-chemotherapy lesions SUV_max_ T/M_max_	Lesions half a year after transplantation SUV_max_ T/M_max_	Lesions one year after transplantation SUV_max_ T/M_max_	Post-transplantation Visit time (months)	Status
1	66	M	IgA-κ	3	2	CR	SUV_max_ 1.9 T/M_max_ 1.1	SUV_max_ 2.1 T/M_max_1.8	SUV_max_ 2.5 T/M_max_ 1.4	/	8.3	CR
2	57	F	IgG-κ	3	3	nCR	SUV_max_ 4.2 T/M_max_ 1.6	SUV_max_ 3.3 T/M_max_ 3.7	/	/	/	Died
3	48	F	IgA-κ	2	1	nCR	SUV_max_ 2.0 T/M_max_ 2.5	SUV_max_ 1.7 T/M_max_ 0.9	SUV_max_ 1.1 T/M_max_ 0.7	SUV_max_ 2.3 T/M_max_ 1.3	18.3	CR
4	46	M	IgG-κ	3	2	CR:	SUV_max_ 4.5 T/M_max_ 2.1	SUV_max_ 0.8 T/M_max_ 0.8	SUV_max_ 2.3 T/MV_max_ 1.0	/	11.8	CR

## DISCUSSION

In this study, we used both SUV_max_ and T/M_max_ to assess ^18^F-FDG uptake in lesions from 98 newly diagnosed MM patients with ^18^F-FDG PET/CT scans. Both indexes performed well at differentiating MM bone lesions from healthy controls. Although T/M_max_ had a low sensitivity of 65.3% in finding out lesions, its specificity was 100%, indicating that T/Mmax is also better than SUV_max_ for judging treatment response. Subsequent studies by our group also confirmed that T/M_max_ had a similar role to SUV_max_ in assessing treatment response, PFS and OS; however, as the cases in the study in limited, more case studies are required to confirm this conclusion.

Our study demonstrated that ^18^F-FDG PET/CT has greater sensitivity in detecting bone lesions (in patients with newly diagnosed MM) compared with systemic bone X-rays, in identifying extramedullary infiltration lesions, and that it is a valuable tool for the diagnosis of MM; these results are in agreement with previous studies[1–3, 7–11].

In addition to demonstrating the increased sensitivity of ^18^F-FDG PET/CT in detecting bone lesions, ^18^F-FDG PET/CT could also be used to evaluate prognosis. One study found that if patients had lesion with SUVmax> 4.2 at the onset of the disease, he or she had shorter DFS and OS. The result indicates that the metabolic profile in bone lesions is closely related to prognosis [22]. From a clinicopathological perspective, ^18^F-FDG uptake in lesions was associated with tumor burden and tumor cell mutations of P53. Clinically, M protein levels, the proportion of tumor cells, β2-MG, and LDH are commonly used to assess disease burden in MM patients. Our research find that high M protein levels, a proportion of plasma cells > 20% in bone marrow smear, β_2_-MG > 3.5mg/L, hypercalcemia at the onset of disease, and increased LDH were associated with increased ^18^F-FDG uptake in MM patients. This interesting finding indicates ^18^F-FDG uptake in bone lesions is related to tumor burden, and it may have more appliances than just diagnosis. Moreover, younger age at diseases onset, patients have free immunoglobulin light chains circulating in serum, patients with extramedullary infiltration, patients with high-risk molecular features and poor karyotype were indicators of poor prognosis independent of tumor burden in MM patients.

Another discovery in surprise is that we found several influence factors of PET/CT scan which were not mentioned by others before. A series of studies indicated a correlation between high ^18^F-FDG uptake and the severity of MM [21]. Opposite to our expectation, we found patients with lower hemoglobin levels had lower ^18^F-FDG uptake in lesions. Anemia is a common complication of MM, which means greater tumor burden and poor prognosis in patients. However, our study showed low hemoglobin levels and red blood cell counts were associated with low ^18^F-FDG uptake in MM patients. The metabolism of lesions is reportedly affected by hemoglobin levels and red blood cell counts on PET/CT scans using ^15^O-deoxyhemoglobin as the tracer [21]. Similarly, ^18^F-FDG PET/CT uses glucose metabolism as the tracing target, and red blood cells are one of the most important sites of glucose metabolism; we therefore speculate that hemoglobin levels and red blood cell counts could affect ^18^F-FDG uptake in lesions, in which case ^18^F-FDG uptake might be underestimated in patients with severe anemia. Physicians should therefore take into account hemoglobin levels and red blood cell counts when interpreting ^18^F-FDG uptake in lesions of MM patients in clinical practice.

With regards to MM treatment response evaluation, PET/CT also has practical value: 34 MM patients with similar M protein levels showed no differences in the dynamic radiological examination, ^18^F-FDG uptake showed its priority in detecting minimal tumor residual changes in continued treatment. We even detected metabolically active bone lesions in certain MM patients who had achieved CR by ^18^F-FDG PET/CT, and found that ^18^F-FDG uptake could be further decreased in these lesions with continued treatment. This demonstrates the value of using ^18^F-FDG PET/CT for treatment response evaluation, even beyond CR. We also demonstrated that MM patients with ^18^F-FDG uptake that reduced to normal after chemotherapy had longer PFS and OS and that PET/CT has certain value in evaluating short- and long-term therapeutic response. Tumor burden might be underestimated if treatment response was estimated based on the maximum ^18^F-FDG uptake of a single lesion. And in these patients, the appliance of Whole body MRI may show its priority[22].

We attempted to seek early parameters indicative of clinical recurrence based on the change in ^18^F-FDG uptake in MM patients. However, the increased ^18^F-FDG uptake after treatment was not necessarily associated with recurrence based on the current data. Only 1 of 4 MM patients who had increased ^18^F-FDG uptake after treatment had clinical recurrence, and the remaining 3 patients were still in CR at last follow-up. This result indicates that increased ^18^F-FDG uptake in lesions in MM patients might be related to hemoglobin levels and infection, and cannot simply be used for predicting disease progression or recurrence.

In summary, the new parameter T/M_max_ should be applied to interpret lesions in MM patients and the impact of hemoglobin levels should be taken into consideration when interpreting the results from ^18^F-FDG PET/CT. Accurate interpretation of ^18^F-FDG results may greatly aid MM diagnosis, and evaluation of prognosis and treatment response. However, further study is required to establish the significance of this test for predicting disease recurrence.
